# The association between sexual function and body image among postmenopausal women: a cross-sectional study

**DOI:** 10.1186/s12905-021-01549-1

**Published:** 2021-12-07

**Authors:** Soheila Nazarpour, Masoumeh Simbar, Mobina Khorrami, Zahra Jafari Torkamani, Reyhaneh Saghafi, Hamid Alavi-Majd

**Affiliations:** 1 Department of Midwifery, Chalous Branch, Islamic Azad University, Chalous, Iran; 2 Midwifery and Reproductive Health Research Center, Department of Midwifery and Reproductive Health, School of Nursing and Midwifery, Shahid Beheshti University of Medical Sciences, Vali-Asr Avenue, Cross of Vali-Asr and H Highway, Opposite to Rajaee Heart Hospital, 1996835119 Tehran, Iran; 3 Department of Midwifery and Reproductive Health, School of Nursing and Midwifery, Shahid Beheshti University of Medical Sciences, Tehran, Iran; 4 Department of Biostatistics, School of Paramedicine, Shahid Beheshti University of Medical Sciences, Tehran, Iran

**Keywords:** Menopause, Sexual function body image

## Abstract

**Background:**

Postmenopause physiological changes may alter body image (BI) during the postmenopausal period of life. Body image dissatisfaction may have negative effects on the sexual function of women. The present study aims to assess the relationship between body image and sexual function during the postmenopausal period.

**Methods:**

This cross-sectional study was performed on 231 postmenopausal women who were menopausal during the recent 5 years, aged > 40 years old, and referring to health centers in Tehran-Iran. The participants were recruited using a multi-stage sampling method. Data were collected using 3 questionnaires including socio-demographic, Fisher’s Body Image, and female sexual function index (FSFI). Data were analyzed using SPSS 24 and multiple regression, Mann–Whitey, Pearson, and Spearman correlation tests.

**Results:**

Two hundred thirty-one postmenopausal women aged 52.53 ± 5.32 (mean ± SD) years old participated in the study. The mean and standard deviation of FSFI and BI of the women were respectively 19.59 ± 11.11(ranges 1.2–36) and 155.43 ± 37.09 (ranges from 46 to 230). According to FSFI, 62.8% of female sexual dysfunction (FSD) was reported. There were significant correlations between scores of total and all domains of FSFI with scores of total and all dimensions of BI. There was also a positive correlation between education and family income of the women and a negative correlation between age of husband and duration of menopause with their sexual function. Significant relationships were also shown between the women's and the husbands' jobs with the total score of women's sexual function. According to the multiple linear regression model, BI was a significant predictor of sexual function in postmenopausal women.

**Conclusion:**

Body image is effective on the sexual function of postmenopausal women. Therefore, body image is necessary to be considered in future postmenopausal health promotion programs.

## Background

Menopause is defined as the permanent cessation of menstruation that results from the loss of ovarian function and therefore represents the end of a woman's reproductive life [1]. Along with the gradual decrease in estrogen and progesterone, which leads to complete ovarian failure and menopause, women experience physical, psychosocial, or sexual changes [2]. Perimenopausal hormonal fluctuations cause women to be exposed to various physical and mental disorders [3]. By the year 2030, there will be 1.2 billion menopausal and postmenopausal women in the world [4].

Menopause is one of the factors affecting sexual function in women [5]. Most middle-aged women (approximate age 40–60) are sexually active and sex is important to them [6], however, several studies showed negative changes in female sexual function in middle age [7, 8].

Sexual function is the result of an interaction between vascular, neurological, and hormonal factors and also is influenced by personal and interpersonal factors, social norms as well as cultural and religious values [9]. Sexual dysfunction is defined as a clinically significant disorder in a person's ability to respond sexually or experience sexual pleasure [10]. Many women experience sexual dysfunction during menopause and the prevalence of female sexual dysfunction (FSD) among postmenopausal women is reported between 25 and 85.2% [11, 12]. A study demonstrated the prevalence of 61% FSD among postmenopausal women in Iran [13].

Postmenopausal female sexual dysfunction is a complex disorder that has many causes [1]. Reasons for these negative changes in sexual function may include biological (e.g., decreased sex hormones), psychological (e.g., the development of mood symptoms), interpersonal (e.g., sexual dysfunction in the sexual partner), and socio-cultural (e.g., negative gender stigma in older women) factors [1, 8, 14].

Menopause is a transitory biological phenomenon, but it also marks a social passage, the significance of which varies in different cultures. It has often been regarded as a negative reinforcement of the aging process and loss of sexual attractiveness [15]. Therefore, Cultural and social context may influence how women perceive and experience sexual function during menopause [16]. A qualitative study in Iran demonstrates many Iranian women perceived menopause as the end of their sexual life [17]. A study also found that the majority of postmenopausal women pay less attention to their sexual wellbeing and consider limitations for their sexual activities [18]. Another study also showed a relationship between socio-demographic characteristics such as ethnicity and body mass index with sexual function among middle-aged Iranian women [19].

During the postmenopausal period, many women experience changes in their body, including increasing body mass index, changes in sexual organs such as sagging breasts, vaginal dryness and atrophy, and appearance of facial wrinkles or excess hair [8, 20] that may lead to body image dissatisfaction and mental problems [21]. Body image is a multidimensional, subjective and dynamic concept that encompasses a person’s perceptions, thoughts, and feelings about their own body. It is one of the main elements of an individuals' personality [22]. These aging-associated physiological changes make the female body away from the thin-young-ideal social norm for beauty. In addition, aging modifies the life priorities and psychological status of postmenopausal women that may affect their body image throughout their lives [23]. These potentially effective factors on women's body image can in turn affect their sexual satisfaction and function [8]. There are a few studies about the body image of middle-aged women. It seems to make positive body image may improve sexual satisfaction and self-confidence of middle-aged women [8, 24].

In Iran, there are over 8.5 million middle-aged women (aged 40–65 years) and more than 14.5% of the total population are women over 40 years [25]. It is estimated that by 2022, about 5 million women will live in menopause in Iran [26]. Respecting the developing population of menopausal women and so essentiality for planning menopausal health program as well as high prevalence of sexual dysfunction and limited studies about the possible association of body image with sexual function among postmenopausal women, this study aimed to determine the correlation between body image and sexual function in postmenopausal women.

## Methods

### Study design

This study was a descriptive cross-sectional correlation study on 231 eligible postmenopausal women who were recruited from attendees to the health centers affiliated with Shahid Beheshti University of Medical Sciences in Tehran, Iran.

The inclusion criteria were: natural menopause in the recent 5 years, age > 40, married and sexually active. The exclusion criteria were: history of medical physical and psychological diseases, occurrence of severe stress such as an accident or losing family members during the last 3 months, taking medicines containing phytoestrogens or estrogen and progesterone, and impotence husband.

### Sample size

The minimum sample size was calculated to be 194, using the following formula for calculating correlation studies as below:

- The standard normal deviate for α = Zα = 1.96
- The standard normal deviate for β = Zβ = 0.84
- C = 0.5 * ln[(1 + r)/(1 − r)] = 0.2027
- Total sample size = N = [(Zα + Zβ)/C]2 + 3 = 194

Considering 20% probability of loss 231 samples were recruited.

### Sampling

A multi-stage sampling method was performed for the recruitment of the subjects of the study. In the first stage, eight health centers were randomly selected by the randomization option of Excel from four geographic districts of Tehran. Then quota sampling was performed to recruit menopausal women with inclusion criteria in the selected centers (28 samples from each selected 6 centers in north, east and west districts; and 63 samples from the two centers in south district). The eligible participants complete the written consent and then the questionnaires were filled up by an interview.

### Measures

Three questionnaires were used for data collection including a researcher-made Socio-demographics questionnaire, the Fisher body image questionnaire [27], and the Rosen et al. Female Sexual Function Index (FSFI) questionnaire [28].

*The socio-demographics questionnaire* The questionnaire contains 22 questions including demographic, socio-economic, and fertility history questions.

*Female sexual function index (FSFI)* The questionnaire consists of 19 questions and measures women's sexual function in six domains including (desire, arousal, lubrication, orgasm, satisfaction, and pain). The minimum and maximum of all domains except desire are zero to 6.0 and for desire 1.2–6.0. The total score of scale is obtained by the addition of the scores of the six domains. The total score of FSFI is between 1.2 and 36, and higher scores show better sexual function. FSFI assess the sexual function of women during the past 4 weeks. A total score equal to or less than 26.55 is considered as FSD [28, 29]. The reliability and validity of the Persian version of the FSFI questionnaire were confirmed by Mohammadi et al. [29] and Fakhri et al. [30]. Nazarpour and colleagues also showed Cronbach's alpha 0.938 and Intra-class correlation coefficient 0.997 [31].

*The fisher body image questionnaire* It was developed by Fisher in 1970 and includes 46 items. The items are scored from 1 to 5 for very dissatisfied, dissatisfied, average, satisfied, and very satisfied, respectively. The total score ranges from 46 to 230 and high scores show positive body image. The dimensions include: head and face (12 items), upper limbs (10 items), lower limbs (6 items) and the other 18 items measure the subject's attitudes toward general body characteristics [32]. The validity of the test was assessed in Iran [32, 33]. In a study by Nazarpour and Khazai [34], the reliability of the Fisher body Image questionnaire was shown by α-Cronbach, Spearman, Guttman Split-half coefficient of 0.918, 0.861, and 0.861, respectively. Test–retest Pearson correlation coefficient 0.84 was also calculated in the previous studies [32, 33].

### The procedure of the study

After assessment of the inclusion criteria of the women, a written consent form was obtained and then the questionnaires were completed using face-to-face interviews with the eligible participants by the interviewers who were trained by the researchers. Face-to-face interviews were used to improve the accuracy of responses to the questions as the researchers predicted that some of the respondents would be illiterate.

### Statistical analysis

The data were analyzed using the SPSS version-24 statistics software. The correlation coefficients were calculated by using the Pearson correlation test for quantitative variables with normal distribution and the Spearman correlation test for ranked and non-normal variables. Mann–Whitey test was also used to compare groups. Multiple linear regression was used to determine the predictors of sexual function.

Our assumption for the multiple linear regression model was that sexual function (FSFI) was related to body image. In multiple linear regression, the total FSFI score was considered as the dependent variable, and the total score of body image was considered as the main independent variable. Age, age of husband, duration of menopause, the job of women and her spouse, educational level of women and her spouse, the adequacy of Monthly income were considered as the potential confounding variables and so included in regression models. These variables were shown to be associated with sexual function [12].

The level of significance was set at *P* less than 0.05.

## Results

Two hundred and thirty-one postmenopausal women with an average age of 52.53 ± 5.23 (Mean ± SD) years old participated in the study. Age of menopause and duration of menopause were 50.28 ± 4.83 and 2.22 ± 1.49 years, respectively. Socio-demographic and obstetrics characteristics of women are shown in Table 1.

**Table 1:** Socio-demographic characteristics of the postmenopausal women (n = 231)

Variables	Mean ± SD/n(%)
Age (years)	52.53 ± 5.23
Age of husband (years)	56.96 ± 7.82
Duration of menopause (years)	2.22 ± 1.49
Age of menopause (years)	50.28 ± 4.83
Gravida	3.42 ± 1.52
Parity	2.96 ± 1.48
Live child	2.88 ± 1.32
Still birth	0.07 ± 0.33
Education	
Illiterate	10 (4.3)
The literacy level of reading and writing	36 (15.6)
Under diploma	48 (20.8)
Diploma	80 (34.6)
Graduate diploma	10 (4.3)
Bachelor	33 (14.3)
Master of science (M.Sc.)	10 (4.3)
Doctorate	3 (1.3)
Education of husband	
Illiterate	3 (1.3)
Literacy level of reading and writing	23 (10.0)
Under diploma	32 (13.9)
Diploma	75 (32.5)
Graduate diploma	24 (10.4)
Bachelor	59 (25.5)
Master of science (M.Sc.)	8 (3.5)
Doctorate	4 (1.7)
Woman’s job	
Housewife	154 (66.7)
Employed	57 (24.7)
Retired	20 (8.7)
Husband’s job	
Employed	147 (63.6)
Retired or unemployed	83 (35.8)
Homeownership status	
The owner	149 (64.5)
Non-owner	82 (35.5)
The adequacy of family monthly income	
Inadequate	94 (40.7)
Adequate	137 (59.3)

^a^ Menopause is defined as the time when there are no menstrual periods for 12 consecutive months

The mean score for FSFI was 19.59 ± 11(ranges 1.2–36). FSD was observed in 62.8% of postmenopausal women. Mean scores of FSFI and its domains are presented in Table 2.

**Table 2:** The mean and standard deviation of postmenopausal women’s scores of body image and sexual function (FSFI)

Variables	Score
	Mean ± SD
*Body image*	
Head and face	41.11 ± 9.87
Upper limbs	34.31 ± 8.40
Lower limbs	20.01 ± 5.59
Overall	60.07 ± 15.00
Total body image	155.43 ± 37.09
*FSFI*	
Desire	3.24 ± 1.45
Arousal	3.00 ± 2.09
Lubrication	3.15 ± 2.27
Orgasm	3.45 ± 2.15
Satisfaction	3.79 ± 2.05
Pain	3.03 ± 2.38
Total FSFI	19.59 ± 11.11

The mean score of body image was 155.43 ± 37.09 (ranges from 46 to 230). Mean scores of body image and its domains are presented in Table 2.

The results of the study demonstrated the positive significant correlations between total score and scores for all domains of sexual function, with the total score and scores of all dimensions of body image (*P* < 0.001) (Table 3).

**Table 3:** Correlations between sexual function (FSFI) and the body image (n = 231) of postmenopausal women

Domains	Body image				
	Head and face	Upper limbs	Lower limbs	Total	Total body image
	r	r	r	r	r
*Sexual function*					
Desire	0.328*	0.392*	0.371*	0.323*	0.366*
Arousal	0.318*	0.368*	0.347*	0.271*	0.331*
Lubrication	0.357*	0.422*	0.436*	0.334*	0.392*
Orgasm	0.328*	0.381*	0.388*	0.301*	0.351*
Satisfaction	0.338*	0.400*	0.409*	0.261*	0.339*
Pain	0.330*	0.387*	0.404*	0.313*	0.370*
Total score	0.367*	0.431*	0.438*	0.327*	0.394*

*P < 0.001; Test: Pearson correlation coefficient

The results showed significant positive correlations between the total score of FSFI and the participant's level of education (*P* < 0.001, r = 0.267), the adequacy of their family monthly income (*P* = 0.021, r = 0.154). Also, significant negative correlations were observed between the total score of FSFI score and the age of the spouse (*P* = 0.032, r = − 0.144) and the duration of menopause (*P* = 0.001, r = − 0.214) (Table 4).

**Table 4:** The correlation between socio-demographic factors and sexual function of the postmenopausal women (n = 231)

Socio-demographic factors	Sexual function (total score of FSFI)	
	*r*^a^	*P*
*Spearmen correlation test*		
Age	*r* = − 0.117	0.081
Age of husband	*r* = − 0.144	0.032
Age of menopause	*r* = − 0.068	0.312
Duration of menopause	*r* = − 0.214	0.001
Duration of marriage	*r* = − 0.095	0.159
Education	*r* = 0.267	< 0.001
Education of husband	*r* = 0.085	0.207
The adequacy of family monthly income	*r* = 0.154	0.021
*Mann–Whitney test*^*b*^		
Woman’s Job	*z* = − 3.479	0.001
Husband’s Job	*z* = − 5.041	< 0.001
Homeownership status	*z* = − 0.756	0.449

^a^*r*: Spearmen correlation was used for data analysis since the data were numerical variables with non-normal distribution; ^b^ Mann–Whitney Test was used for data analysis as the educational level, and the adequacy of family monthly income were ordinal variables

The total score of FSFI of employed people was significantly higher than non-employed people (housewives and retirees) (*P* = 0.001). Also, the women with employed husbands had significantly higher FSFI scores (*P* < 0.001) compared to the women with retired or unemployed husbands (Table 4).

The model of multiple linear regression predicts 25% of the total score of female sexual function of postmenopausal women. The results of linear multiple regression showed that body image is a potential predictor of sexual function; So that for each unit of increase in the total score of body image, the total score of sexual function increases by 0.09 units (*P* < 0.001). Based on the model, female education (*P* = 0.002), husband's job (*P* < 0.001), were also the potential predictors of postmenopausal women’s sexual function (Table 5).

**Table 5:** Multiple linear regression to assess potential predictors for the menopausal women sexual function

Predictors	Multiple linear regression					
	B	Beta	T	*P* value	95% Confidence Interval for B	
					Lower	Upper
Body Image	0.088	0.289	4.482	< 0.001	0.049	0.127
Job of husband	5.780	0.248	4.000	< 0.001	2.932	8.629
Education^b^	1.451	0.199	3.169	0.002	0.548	2.354

^a^Classification of Job of husband:0. Retired or unemployed, 1. Employed

^b^Classification of Education: 1. Illiterate, 2. Literacy level of reading and writing, 3. Under diploma, 4. Diploma, 5. Graduate Diploma, 6. Bachelor, 7. Master of Science (M.Sc.), and 8. Doctorate

## Discussion

This study aimed to assess the relationship between body image and sexual function in postmenopausal women. This study showed a significant positive correlation between body image and sexual function of postmenopausal women. It also demonstrated positive significant correlations between all domains of sexual function (desire, arousal, lubrication, orgasm, satisfaction, and pain) with all dimensions of body image. The multiple linear regression model also shows for each unit of increase in the total score of body image, the total score of sexual function increases by 0.09 units. Consistent with these results, various studies emphasize the role of body image in various aspects of sexual activity and sexual function of postmenopausal women [8, 35, 36]. In a similar cross-sectional study on middle-aged women by Afshari et al. in Iran, there were significant correlations between body image with the women’s sexual function (FSFI) and all its domains. They demonstrated that women with a positive body image had a higher score of sexual function, comparing to women with a negative body image. In their analysis, the women’s satisfaction with the shape of the body was a predictor of sexual function [35]. However, they used Body Shape Questionnaire (BSQ) for body image measurement. Thomas and colleagues in their qualitative study in Pennsylvania and on 45–60 years middle-aged women explored that women's body image is an important actor for their sexual satisfaction and attractiveness [8]. Although the study by Thomas et al. was not conducted on middle-aged women, and not specifically on postmenopausal women, the participants were aged in the menopausal age range. Pujols and colleagues also demonstrated a positive association between body image, sexual function, and sexual satisfaction. The study found that several aspects of body image, including weight concerns, physical condition, sexual attractiveness, and mental preoccupation with appearance during sexual activity, predict the sexual satisfaction of women [37]. Also, concentrating on decreasing menopause related physical complications, negative attitudes and feelings, concerns and psychological complications are also recommended for improving sexual function of post-menopausal women [38].

Physical changes, especially weight gain, are common in middle age [8]. Besides, the occurrence of menopause along with the physical and psychological changes may lead to negative feelings about attractiveness and body image. So, it seems menopause can hurt sexual satisfaction as one aspect of sexual function. A study in South Korea on middle age perimenopausal women also demonstrates that menopausal symptoms were significantly associated with a sexual function that may be mediated through their body image, depression, and sexual intercourse [36]. Although this study was performed on perimenopausal women, the results are consistent with our results as we considered the first 5 years of the postmenopausal period of the participants.

Studies have also shown that low body image is associated with high levels of orgasmic disorders [39] and a positive body image and high self-acceptance of one's physical appearance are associated with appropriate orgasm and sexual function [40]. This relationship between sexual function and body image can be due to women’s concentration on their body that may distract their attention from positive sexual feelings and sexual signs of their partner, which in turn may lead to reduced self-efficacy and sexual pleasure. Thus, a negative body image may reduce sexual desire, intimacy with a partner, and sexual response [39].

The finding of the present study also showed a significant positive correlation between FSFI with women's education. The multiple linear regression analysis also showed education as a predictor of FSFI. This result is consistent with similar studies [19, 41–44] that show education as a predicting variable for psychological complications and negative menopausal feelings with the potential negative effects on women's sexual function during menopause. The high awareness that can be gained through higher education can improve women’s attitudes and thoughts about gender, reduce anxiety, and help the women to cope with menopausal changes [45].

The results of the present study showed that there is a significant negative correlation between the age of the spouse and the sexual function of postmenopausal women. This result is consistent with some other studies [12, 41, 43, 46]. This association may be due to hormonal changes due to aging and its consequences on sexual partner sexual function [12], which in turn may affect female sexual function. Contrary to previous studies [12, 45], there was not a significant correlation between women’s menopausal age and sexual function as we recruited women who became menopause during the last 5 years, and so, the average age of the participants was 52.5 years, and this causes the range of age variable limitation for the postmenopausal period, as we wanted to be only limited to postmenopausal period.

There was a significant negative correlation between the sexual function of postmenopausal women and the duration of menopause. There are a few studies that did not confirm this result [47, 48], however, a study showed menopausal women experience more sexual dysfunction during the first year of menopause than second to 5 year, but after 5 years of menopause, more dysfunction is reported with increasing age. This result may be due to the menopausal symptoms caused by the sudden cessation of hormones during the first year of menopause. Menopausal women may gradually adapt to the change and enjoy their sexual life after about a year [49]. This controversy is due to considering the zero to 5 years duration of menopause in our study, so further studies with a focus on the effects of duration of menopause on female sexual function are necessary.

The results of the study indicated a significant positive correlation between the women’s sexual function and their family monthly income. This result is consistent with the results of other studies [12, 42, 45, 50, 51]. It seems high income is associated with high quality of life and less anxiety and depression and so leading to sexual satisfaction [31, 45, 52].

According to the results of the present study, the employment of menopausal women and their husband was associated with improved sexual function. The multiple linear regression analysis also demonstrated their husband’s employment was a potential predictor of sexual function. Although some other studies confirmed that women's employment is associated with better sexual function [42, 44], a study did not confirm it [53]. There was no study on the association of employment of postmenopausal women's husbands with their sexual function, but in the study of Jafarzadeh-Esfahai and colleagues, this relationship was not significant among women of different ages [54].

Therefore, it can postulate that socio-economic factors such as education, employment, and family income are associated with a higher quality of life and body image and sexual function. These factors are also effective on individuals' and societies' perceptions of gender and sexual behaviors [45]. In this accordance, to promote sexual health among postmenopausal women sociodemographic socio-demographic and cultural context should be considered.

### Strengths and limitations

This study was performed on women during the first 5 years of menopause as we aimed to assess the association of menopausal changes on sexual function and not the effects of aging on sexual function. In addition, the study was conducted on a population of postmenopausal women with a normal distribution that reduced participation bias.

A limitation of the present study was that it was a descriptive study and so the results can only show the association of the variables, and not cause and effect relationships. So analytical studies are necessary to show the causative effect of body image on postmenopausal women's sexual function.

The second limitation was the self-reported data gathering about body image and sexual function, and also demographics characteristics that may make self-report bias which means the deviation between the self-reported and true values of the same measure.

Besides, sexual function is a private issue of life and FSFI could be better to be self-completed. However, it was completed by interview by the trained interviewers, because a group of the participants was illiterate or low literate (40.7% under diploma) and besides we want to be sure that the condition for filling the questionnaire is consistent for all subjects, and also to be sure about not losing any response. It should be noted that all participants were explained about the aim of the study and gave us consent to ask the questions. Besides, FSFI is a self-report of sexual function and can be completed by interview.

Furthermore, a cause of sexual dysfunction among postmenopausal women is atrophic vaginitis. However, the aim of the present study was to assess the possible correlation of sexual function and body image. As mentioned before, vaginal atrophy is a common postmenopausal complication caused by estrogen deficiency and is known as one of several suggested causes for sexual dysfunction of postmenopausal women. However, assessment of the causes of each of the two variables (sexual function and body image) were not necessary. In this study condition for participation was their menopausal duration (up to the first 5 years) and no hormone therapy to recruit samples with similar conditions in terms of atrophic vaginitis. Anyway, atrophic vaginitis can be a main cause of sexual dysfunction among postmenopausal women, and solving this problem with systemic or local hormones or lubricants [55] can be recommended to improve the both sexual dysfunction and so body image.

## Conclusion

The present study showed that postmenopausal women's body image is correlated with their sexual function. Since sexual dysfunction is very common among postmenopausal women, health care providers should be aware of the relationship between dissatisfaction with body image and sexual dysfunction, and the treatment of sexual dysfunction should include obtaining information about women's attitudes towards their body image. It seems improvement of postmenopausal women’s body image promotes their sexual function and then sexual satisfaction. Regarding the significant correlation between body image and sexual function of post-menopausal women and according to the prevailing culture of the society, health policies and programs should be developed to help postmenopausal women in accepting and adapting the physical and psychological changes caused by menopause, and so that to prevent hurt to their body image. It should be also emphasized that improving in socio-economic factors such as women's education, employment and income are also associated with promoting female sexual function that should be considered by major health policymakers of the country.

## Data Availability

The datasets used and/or analyzed during the current study are available from the corresponding author on reasonable request.

## Abbreviations

BI: Body image
FSFI: Female sexual function index
FSD: Female sexual dysfunction
SD: Standard deviation
SPSS: Statistical package for the social sciences
